# Exploring the potential role of EPSPS mutations for enhanced glyphosate resistance in *Nicotiana tabacum*

**DOI:** 10.3389/fpls.2025.1516963

**Published:** 2025-02-10

**Authors:** Bingjie Li, Chen Chen, Mengmeng Cui, Yuhe Sun, Jing Lv, Changbo Dai

**Affiliations:** ^1^ Tobacco Research Institute, Chinese Academy of Agricultural Sciences, Qingdao, China; ^2^ Graduate School of Chinese Academy of Agricultural Sciences, Beijing, China

**Keywords:** glyphosate, herbicide resistance, EPSP synthase, tobacco, gene editing

## Abstract

Glyphosate is a widely used non-selective, broad-spectrum, systemic herbicide by interfering with the biosynthesis of aromatic amino acids. However, the emergence of glyphosate-resistant weeds has driven the need for enhanced herbicide resistance in crops. In this study, we engineered two mutant variants of the tobacco EPSPS gene through amino acid substitution (TIPS-NtEPSPS and P180S-NtEPSPS). These mutated *EPSPS* genes were overexpressed in tobacco under the control of CaMV35S promoters. Our results demonstrate that overexpression of TIPS-NtEPSPS significantly enhances glyphosate tolerance, allowing plants to withstand up to four times the recommended dose without compromising their fitness. This research highlights the potential of the TIPS-NtEPSPS mutant to improve herbicide resistance in tobacco, offering a viable approach for effective weed management.

## 1 Introduction

The presence of weeds significantly impacts global crop productivity. The development of herbicide-resistant (HR) crops and the application of herbicides have become cost-effective methods for weed management. Agricultural workers have increased the application of herbicides to control weeds, resulting in residual effects on subsequent crops, such as tobacco, in rotation areas. In mild cases, this affects the normal development and growth of tobacco leaves, leading to reduced yields and quality. In severe cases, it can cause total crop failure. Glyphosate, the most extensively used systemic broad-spectrum herbicide, has revolutionized modern agriculture by controlling weeds in crop fields thereby enhancing global crop productivity (Wang et al., 2014). The shikimate pathway, crucial in plants, fungi, and microbes, features the EPSPS enzyme as a key target for creating glyphosate-resistant (GR) crops (Green, 2014). The first gene to confer glyphosate tolerance in many economically and commercially significant crop species was the naturally occurring glyphosate-insensitive type II AroA enzyme derived from Agrobacterium sp. strain CP4 (Qaim and Kouser, 2013).

The enzyme 5-enolpyruvylshikimate-3-phosphate synthase (EPSPS; EC 2.5.1.19), targeted by the global herbicide glyphosate, catalyzes the synthesis of aromatic amino acids through the shikimate pathway, playing a crucial role in plant metabolism (Smart and Amrhein, 1987; Duke and Powles, 2008). Resistance in various weed species often arises from point mutations in conserved regions of EPSPS, such as Pro 106, Thr 102, and Gly 101 (Sammons and Gaines, 2014; Beres et al., 2020; Baek et al., 2021; Yanniccari et al., 2022). Studies involving the crystal structure analysis of recombinant EPSPS from *Escherichia coli* have identified structural alterations in the mutated enzyme that hinder glyphosate binding, leading to resistance; however, this resistance is primarily inferred from changes in the Michaelis constant (Km) (Healy-Fried et al., 2007; Funke et al., 2009; Sammons et al., 2018; Beres et al., 2020). Class II EPSPS enzymes are primarily utilized to develop glyphosate-tolerant crops that are agriculturally and economically significant (Kahrizi et al., 2007; Chhapekar et al., 2015). Conversely, Class I EPSPS enzymes are susceptible to inhibition by micromolar concentrations of glyphosate and exhibit low affinity for PEP (phosphoenolpyruvate) substrates (Pollegioni et al., 2011). To date, both naturally occurring Class II enzymes and mutated variants of Class I EPSPS have been employed to develop commercial glyphosate-resistant (GR) crops (Funke et al., 2009; Yu et al., 2015). A notable discovery was the T102I and P106S (TIPS) double amino acid substitution in *Eleusine indica*, which provided a glyphosate tolerance level that was 2647 times greater than that of the wild type (WT) and 600 times greater than the single substitution mutant (P106S) (Damgaard and Treebak, 2022).

Efforts to develop glyphosate-resistant genes through mutations at the EPSPS target site have been ongoing for many years. The first mutation-induced GR *EPSPS* gene, *aroA*, was derived from *Salmonella typhimurium* and demonstrated glyphosate resistance when transferred to *E. coli (* Comai et al., 1983). Mutations at positions G96A and A183T in the *E. coli* EPSPS were specifically engineered, and the mutated genes were transferred into canola using Agrobacterium-mediated transformation, allowing the transgenic canola to tolerate a 10 mmol/L glyphosate solution (Kahrizi et al., 2007). E. coli EPSP synthase, a type I enzyme, exhibits glyphosate insensitivity and maintains high affinity for PEP when double mutations T97I and P101S occur simultaneously; such EPSPS variants are typically referred to as TIPS EPSPS (Funke et al., 2006). Using DNA shuffling, Tian et al. (2013) identified an apple EPSPS gene with eight amino acid mutations, with the T101A and T187A mutations playing a crucial role in glyphosate resistance (Tian et al., 2013). This mutated gene, *MdEPSPS* 101/187, was subsequently introduced into rice, resulting in a glyphosate-resistant variety capable of tolerating a 2.5% concentration of glyphosate at 10 L/ha. The first commercial GR maize (GA21) was developed by incorporating a double-mutated maize *EPSPS* gene at T102I and P106S (Sammons and Gaines, 2014). The overexpression of TIPS-OsEPSPS resulted in higher tolerance to glyphosate (up to threefold of the recommended dose) in both controlled and field conditions (Achary et al., 2020). Additionally, precise base editing of conserved amino acids in the EPSPS gene within plant genomes has also successfully produced glyphosate-resistant rice and cassava (Li et al., 2016; Hummel et al., 2018).

Previous studies have obtained highly glyphosate-tolerant tobacco by co-expression of the G2-aroA and gat genes, with tolerant concentrations of 0.2 mmol L^-1^ and 1L ha^-1^ in medium and greenhouse, respectively (Dun et al., 2014). Vennapusa et al. found that expression of several genes with different functions, e.g. by stacking transgenes coding for different detoxification mechanisms and insensitive EPSPS, is a potential approach for developing glyphosate-resistant plants with less residual content (Vennapusa et al., 2022), however, a bloated recombinant vector is the result of introducing large fragments. Here we tried to investigate the potential of genes with point mutations on the enhanced glyphosate tolerance in the model plant tobacco through various types of point mutations in the EPSPS gene using intelligent prediction software, such as AlphaFold. This study focuses on the EPSPS gene in *Nicotiana tabacum* L. (NP_001312842), leveraging previously reported characteristics of glyphosate-resistant EPSPS amino acid sequences. We synthesized the coding region of the tobacco EPSPS gene and introduced two single-base mutations within its conserved domains, altering the amino acids from Thr and Pro to Ile and Ser. The constitutively overexpressed TIPS-NtEPSPS, with a double substitution mutation, conferred significant resistance to higher doses of glyphosate under both controlled and field conditions. Given these promising results, this method shows great potential for implementing TIPS mutations in other elite crops, utilizing either gene transformation or conventional breeding paired with marker-assisted selection to develop glyphosate-tolerant varieties suitable for modern agriculture.

## 2 Results

### 2.1 In silico analysis and identification of glyphosate tolerant mutations

**Figure 1:**
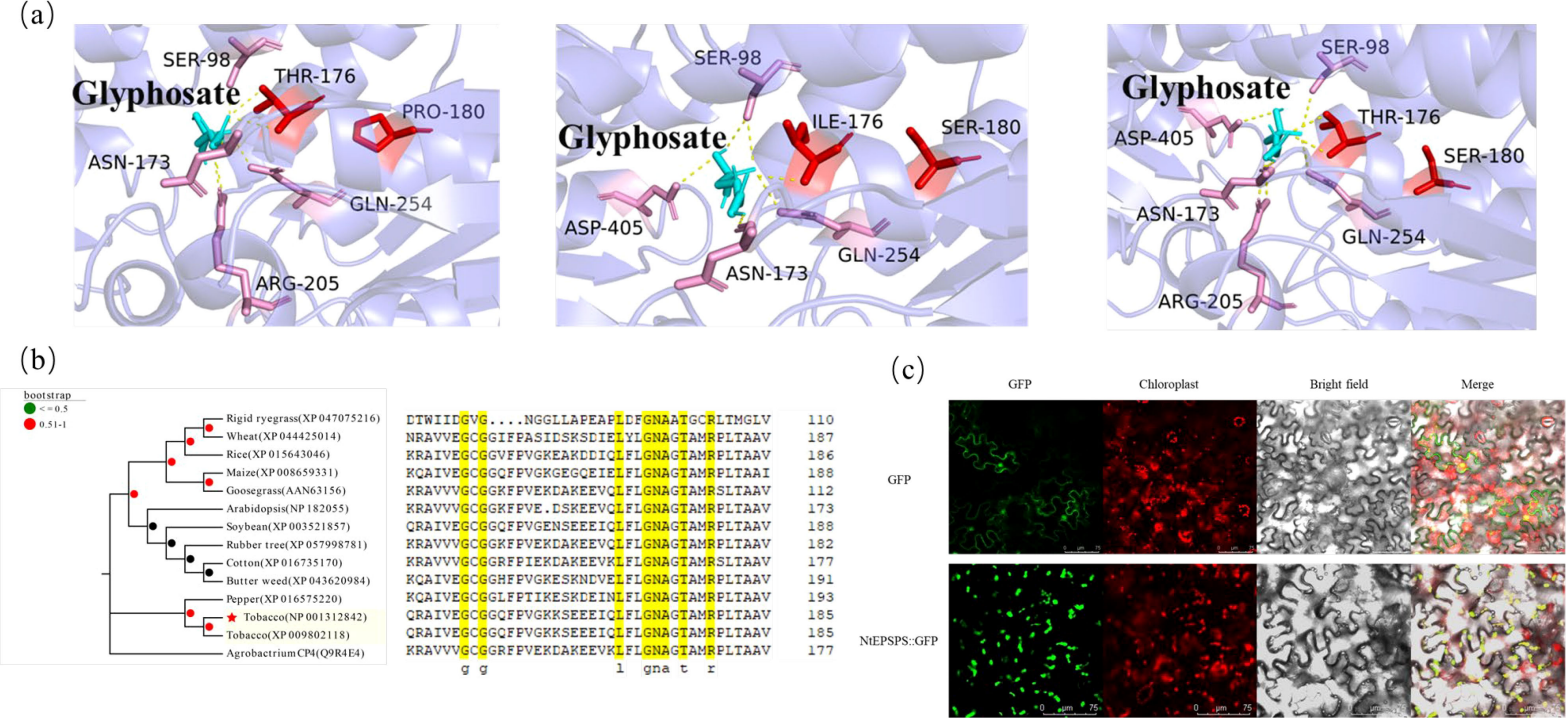
Presents the computational analysis undertaken to identify conserved amino acid residues within EPSPS proteins across diverse organisms, alongside the determination of their phylogenetic relationships and the prediction of protein structures. Additionally, the fluorescent localization of tobacco EPSPS protein is depicted. **(A)** depicts the predicted protein structure highlighting mutation sites and the interaction of glyphosate within the active site of tobacco EPSPS. **(B)** showcases the phylogenetic analysis of EPSPS proteins within plant species. **(C)** illustrates the detection of the empty vector pCAMBIA1305-GFP and the expression vector pCAMBIA1305-NtEPSPS-GFP, with a scale reference of 75 μm.

The overuse of glyphosate has resulted in the evolution of GR weed biotypes. This herbicide selection pressure leads to the accumulation of favorable mutations within the active site of the target enzyme (**Figure 1A**), thereby conferring resistance to glyphosate. Binding energies ≤ -5 kJ/mol indicate good binding, the binding energies of glyphosate with NtEPSPS, TIPS-NtEPSPS and P180S-NtEPSPS were -22.59 kJ/mol, -22.18 kJ/mol and -22.30 kJ/mol, respectively, implying good binding. Homology searches, multiple sequence alignments and phylogenetic analysis of protein sequences from various organisms revealed that the tobacco EPSPS enzyme shared 82% to 90% sequence similarity with other class I plant EPSPS, and only 12% with Agrobacterium-CP4 class II EPSPS protein (**Figure 1B**; **Supplementary Table S1**). Further, the sequence analysis of plant EPSPS also revealed that the amino acids G-175 and T-176 (in tobacco EPSPS) were well conserved among the monocots and dicots (**Figure 1B**). Subcellular localization results indicated that the NtEPSPS protein is localized in the chloroplasts (**Figure 1C**).

### 2.2 Systematic construction and molecular analysis of tobacco plants overexpressing modified EPSPS genes

**Figure 2:**
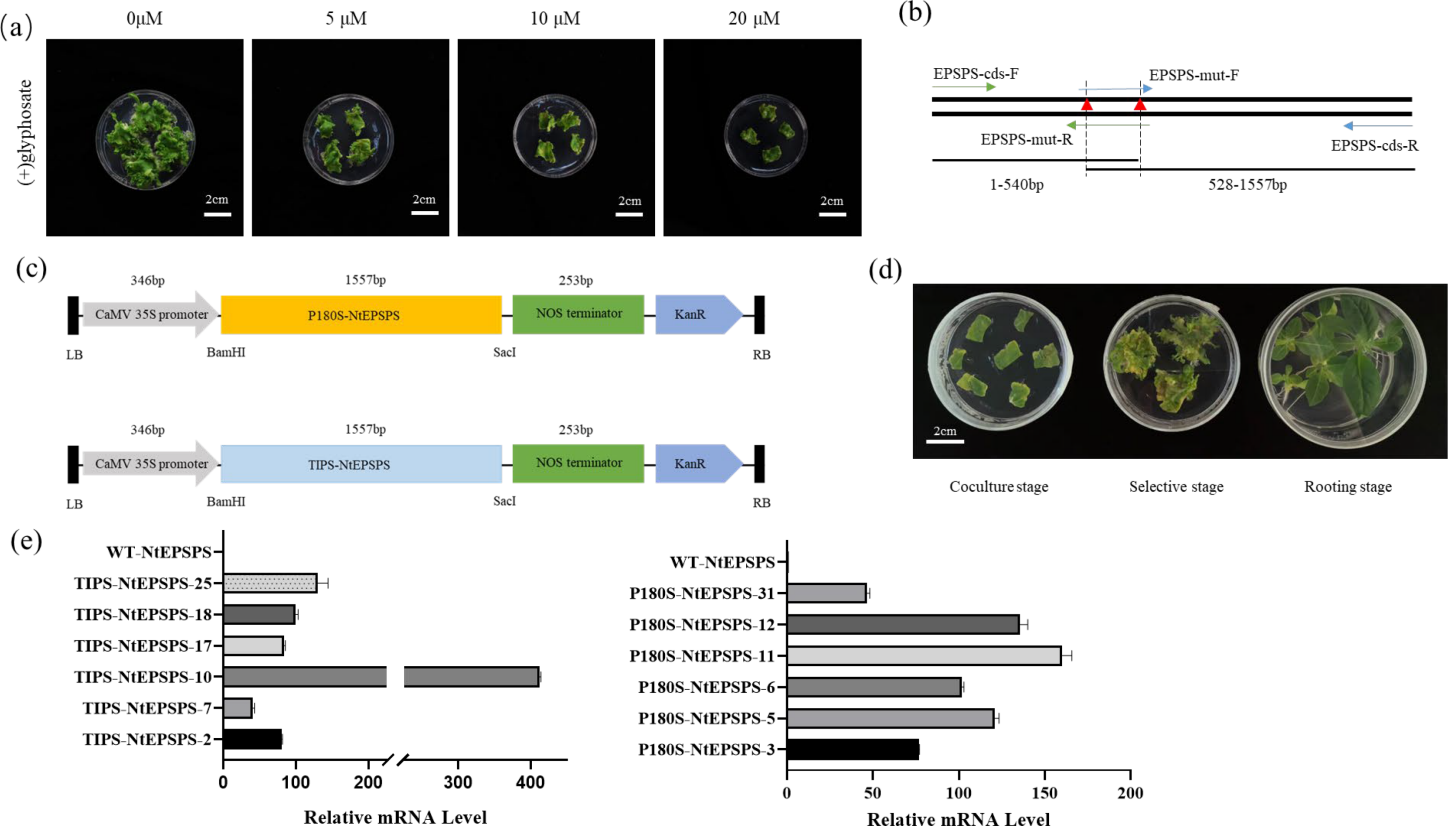
Systematic construction of a plant expression cassette and subsequent resistance analyses of corresponding transgenic materials.**(A)** Assessment of glyphosate resistance in non-transgenic tobacco plants, with photographs captured after 30 days of cultivation on medium containing glyphosate concentrations ranging from 0 to 20 μM. **(B)** Introduction of point mutations into the tobacco EPSPS gene via PCR-mediated techniques. **(C)** Introduction of single (P180S-NtEPSPS) or double (TIPS-NtEPSPS) mutations into the tobacco EPSPS gene (NtEPSPS) through PCR amplification, utilizing primers EPSPS-Mut-F and EPSPS-Mut-R containing modified nucleotide sequences and restriction enzyme sites. The tobacco single-mutant (P180S-NtEPSPS) and double-mutant (TIPS-NtEPSPS) EPSPS variants were expressed under the control of the CaMV 35S promoter. **(D)** Stages involved in the acquisition of genetically transformed seedlings, including coculture, selection, and rooting phases. **(E)** Assessment of the transcriptional levels of overexpressed NtEPSPS (P180S-NtEPSPS and TIPS-NtEPSPS) in tobacco leaves, WT-NtEPSPS is used as CK.

Evaluating glyphosate resistance in non-transgenic tobacco plants is instrumental for subsequent identification of resistance in transgenic materials. When the glyphosate concentration exceeds 10 µM, differentiation of tobacco callus tissue is completely inhibited (**Figure 2A**). In accordance with the molecular docking predictions displayed in **Figure 1A**, we decided to overexpress the mutated P180S-NtEPSPS and TIPS-NtEPSPS (**Figures 2B, C**) genes under the regulation of CaMV 35s promoter systems (**Figures 2B, C**) because EPSPS is a component of the shikimate pathway and synthesizes a variety of metabolites, including essential aromatic amino acids. This strategy aims to enhance glyphosate tolerance and improve the physiological of the overexpressing plants. Two distinct phenotypes of overexpressing plants were generated via agrobacterium-mediated transformation of tobacco leaf tissues (**Figure 2D**). High-expression (≥100-fold) T1 (P180S-NtEPSPS-10/25 and TIPS-NtEPSPS-11/12 progeny were selected for further experimentation (**Figure 2E**).

### 2.3 Physiological characterization of the overexpressed tobacco lines

**Figure 3:**
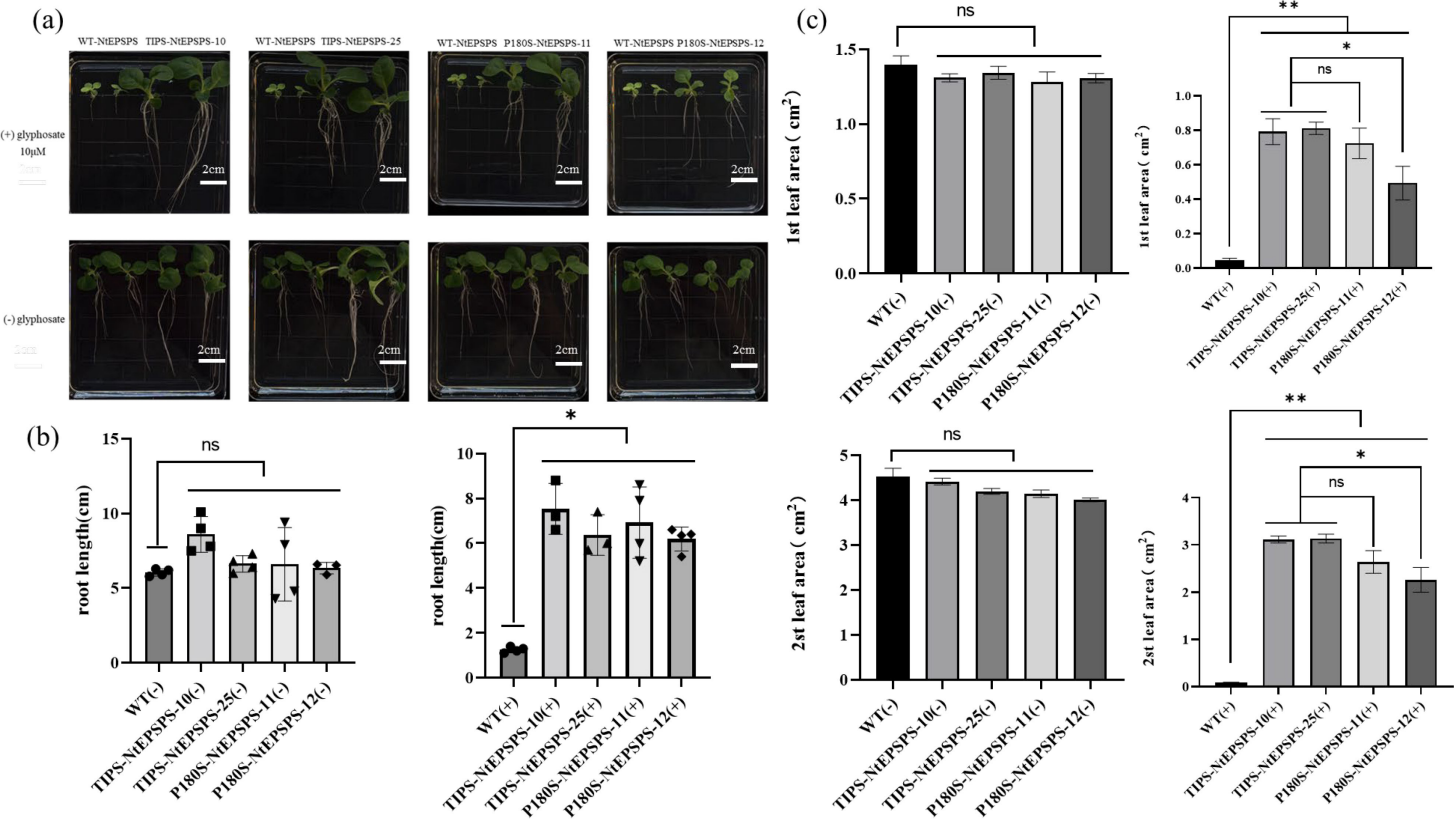
Effects of Glyphosate on Seedling Germination and Growth. **(A)** compares the growth responses of wild type (WT), TIPS, and P180S seedlings to 10 μM glyphosate in the culture medium. includes measurements of root lengths and leaf areas in plants overexpressing these genes, with data shown for varying concentrations of glyphosate. The glyphosate concentrations were found to significantly inhibit (*P ≤ 0.05, **P ≤ 0.01) both root length and leaf area in TIPS and P180S lines compared to their respective WT controls. The study was conducted with five biological replicates and each experiment was repeated three times. The “ns” symbol indicates a non-significant difference **(B, C)**.

We used homozygous and stabilized T1 transgenic tobacco lines for all physiological assays. For the seedling growth experiments, seeds from wild-type (WT) and various T1 transgenic tobacco lines expressing P180S-NtEPSPS single mutant and TIPS-NtEPSPS double mutant were surface sterilized and placed directly on MS media, either without or with 10 µM glyphosate in Petri dish. The germinated seeds were incubated for 15 days in a growth chamber under controlled environmental conditions (25°C, 12:8 h light/dark photoperiod). Plant growth is photographed and growth metrics are noted. The WT tobacco lines exhibited significantly less growth than their mutant counterparts when exposed to glyphosate (**Figure 3A**). The WT tobacco seeds either failed to germinate or suffered severe growth inhibition, appearing yellow leaves in the presence of 10 µM glyphosate. In contrast, both TIPS-NtEPSPS tobacco lines (TIPS-NtEPSPS-10; TIPS-NtEPSPS-25) demonstrated vigorous growth even at glyphosate concentrations that were toxic to WT tobacco. The glyphosate-treated T1 tobacco lines showed growth comparable to untreated WT seedlings in terms of root and shoot development as well as leaf color (**Figure 3A**). These data indicate that the expression of mutant tobacco NtEPSPS lines carrying mutations (T176I and P180S) conferred tolerance to glyphosate toxicity at levels detrimental to WT tobacco plants.

Furthermore, the single mutant (P180S)-overexpressed tobacco lines were also assessed for the level of glyphosate tolerance. As shown in **Figure 3B**, the root length of WT tobacco in the presence of 10 µM glyphosate was significantly inhibited compared to WT plants grown in the absence of glyphosate treatment. In contrast, P180S-NtEPSPS tobacco lines (P180S-NtEPSPS-12) exhibited limited root development and stunted shoot development with moderately green shoots. P180S-NtEPSPS tobacco lines (P180S-NtEPSPS-11) performed better. The tolerance to glyphosate exhibited by the P180S-NtEPSPS plants was marginal (**Figure 3A**). When compared to the WT tobacco EPSPS, the glyphosate tolerance capability of the single-mutant(P180S) variant of tobacco EPSPS exhibited slight improvement and the tolerance was substantially lower than that of the double-mutant (TIPS) variant plants (**Figures 3B, C**). Both types of mutants increased root length by 395.1%-502.4% and leaf area by 960.75%-3524.28% following dichloroquinolinic acid application as compared to WT. In comparison with P180S-NtEPSPS, root length and leaf area of TIPS-NtEPSPS increased by 21.75% and 64.08%, respectively. Collectively, these data suggest that overexpression of double-mutant variants (T176I and P180S) of the EPSPS protein in tobacco enhances tolerance to glyphosate at high dosages (**Figures 3B, C**).

**Figure 4:**
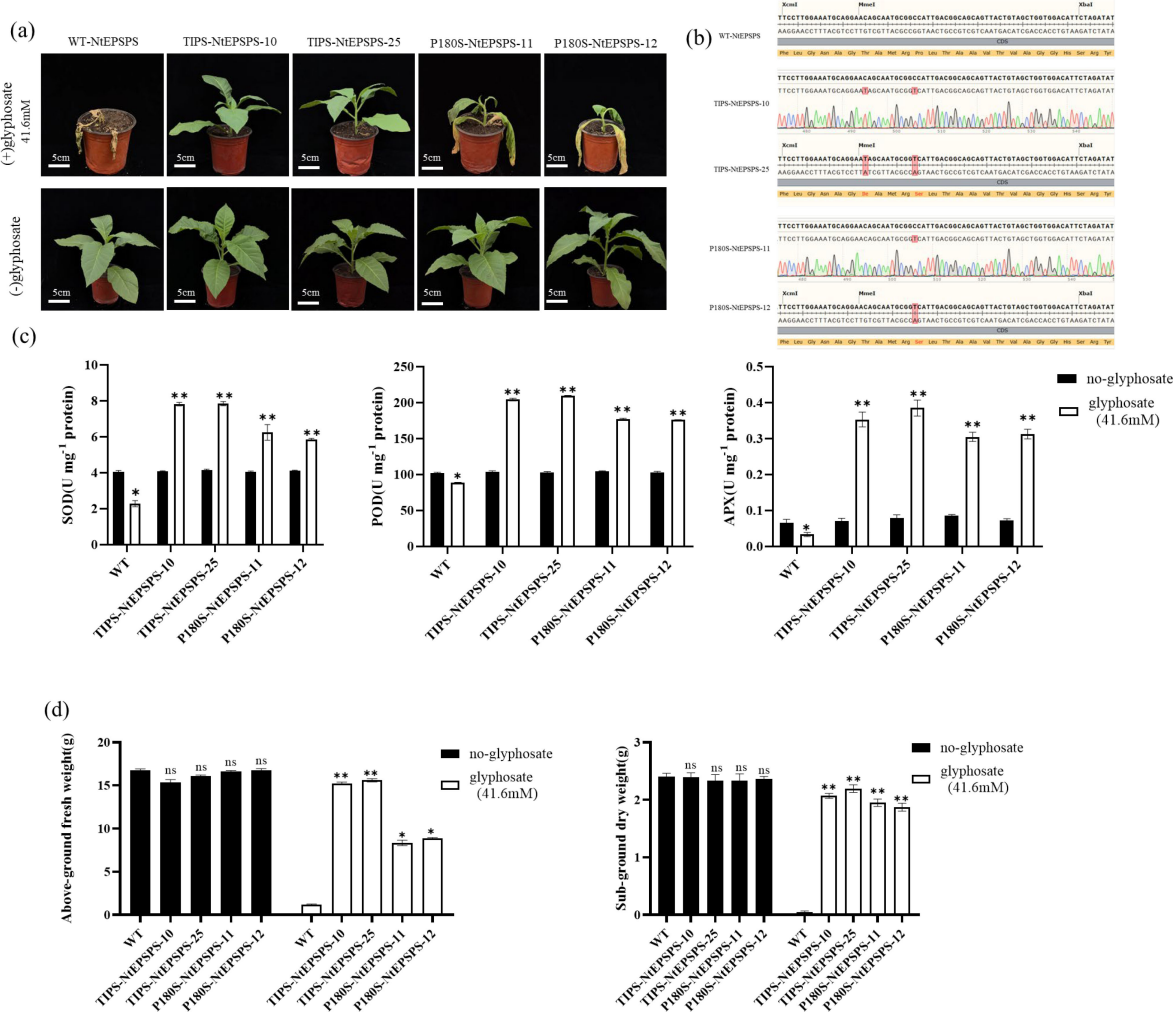
Evaluation of Glyphosate Effects on WT, TIPS, and P180S Plant Lines and Antioxidant Enzyme Activities in Tobacco Leaves. **(A)** Various concentrations of glyphosate (0 mM and 41.6 mM) were applied to WT, TIPS, and P180S plant lines via foliar spray. The TIPS and P180S lines exhibited tolerance and continued growth even under high glyphosate concentrations, with TIPS lines showing superior growth, while WT plants perished under the same conditions. **(B)** Representative profiles depicting the overexpression in TIPS and P180S plant lines. **(C)** Activities of antioxidant enzymes including Superoxide Dismutase (SOD), Peroxidase (POD), and Ascorbate Peroxidase (APX). The glyphosate treatments significantly increased (*P ≤ 0.05, **P ≤ 0.01) the activities of these enzymes **(D)** The fresh and dry weights of TIPS and P180S lines compared to their respective WT controls. The “ns” symbol denotes non-significant results.

To assess the impact of a commercially available glyphosate formulation (41% glyphosate, Roundup Ready, Monsanto) on the growth of wild-type (WT) and various transgenic tobacco lines (P180S-NtEPSPS, TIPS-NtEPSPS), seedlings were cultivated in soil pots. Fifty-day-old seedlings of all lines were sprayed with glyphosate at a concentration of 41.6 mM. The growth and morphological features of these plants were then systematically documented until seed maturation. **Figure 4A** shows that the growth rates of WT, P180S-NtEPSPS and TIPS-NtEPSPS tobacco lines were similar under glyphosate-free conditions. However, exposure to 41.6 mM glyphosate was lethal to WT plants, leading to death within 7 days after application. Although P180S-NtEPSPS plants continued to grow at this concentration, their growth was significantly hindered. In contrast, TIPS-NtEPSPS plants demonstrated obvious glyphosate tolerance showing no adverse effects on growth. Sequencing results indicated that the tested T1 lines were homozygous (**Figure 4B**). These glyphosate-treated TIPS-NtEPSPS plants maintained normal growth and morphological characteristics throughout their development, akin to untreated WT plants.

To elucidate the potential mechanisms behind this enhanced tolerance, we measured the activities of key antioxidant enzymes, including superoxide dismutase (SOD), peroxidase (POD), and ascorbate peroxidase (APX) in both WT and transgenic lines under glyphosate (41.6 mM) and no-glyphosate conditions. Glyphosate treatment significantly increased the activities of all three enzymes in the transgenic lines compared to the no-glyphosate treatment. Specifically, SOD activity was markedly higher in the transgenic lines TIPS-NEPSPS-10, TIPS-NEPSPS-25, P180S-NEPSPS-11, and P180S-NEPSPS-12 when treated with glyphosate (*P ≤ 0.05, **P ≤ 0.01) (**Figure 4C**). Similarly, POD and APX activity was significantly elevated in these transgenic lines under glyphosate treatment. These findings suggest that the transgenic lines may enhance their antioxidant enzyme activities to cope with glyphosate-induced oxidative stress.

Additionally, we also assessed the effect of glyphosate on above-ground fresh weight and sub-ground dry weight in WT and transgenic lines (TIPS-NEPSPS-10, TIPS-NEPSPS-25, P180S-NEPSPS-11, P180S-NEPSPS-12). Glyphosate treatment significantly reduced the above-ground fresh weight in WT plants, while transgenic lines maintained higher fresh weights under glyphosate stress (*P ≤ 0.05, **P ≤ 0.01) (**Figure 4D**). Similarly, sub-ground dry weight was significantly reduced in WT plants under glyphosate treatment, whereas transgenic lines exhibited significantly higher dry weights compared to WT (*P ≤ 0.05, **P ≤ 0.01). Seedling growth retardation was positively correlated with the reduction of antioxidant efficiency under herbicide stress (Ramzan et al., 2022). These results indicate that the transgenic lines possess enhanced tolerance to glyphosate, likely due to increased antioxidant enzyme activities.

Glyphosate residues were measured in both the wild type (WT) and the overexpressed transgenic tobacco lines (TIPS-NEPSPS and P180S-NEPSPS). The analysis showed that glyphosate residues were significantly higher in WT plants compared to the transgenic lines under glyphosate treatment (**Table 1**). The reduced glyphosate residues in the transgenic lines suggest that the overexpression of NEPSPS may enhance glyphosate metabolism or exclusion, thereby contributing to increased glyphosate tolerance.

**Table 1:** Glyphosate concentrations in all parts of WT, TIPS, and P180S plant lines.

Sample	Analytes(mg/kg)	Treatment (mM)	Methods	Days (d)	Part	LOQ	Results
WT	Glyphosate	0	SS/CHN/SOP/4086-01	14	All plant	0.1	<0.1
WT		40.16					27.34 ± 6.34a
TIPS-NtEPSPS-10							16.00 ± 3.55b
TIPS-NtEPSPS-25							17.00 ± 1.63b
P180S- NtEPSPS-11							21.3 ± 3.29ab
P180S- NtEPSPS-12							22.6 ± 1.69ab

Data presented as means ± SE for n = 4. Different letters represent statistically significant differences at P ≤ 0.05 as determined by the Duncan test.

## 3 Discussion

Efforts to develop numerous EPSPS-tolerant mutant variants through site-directed mutagenesis have been undertaken. However, not all mutations have successfully produced GR crops. Additionally, the cultivation of transgenic crops has sparked ethical debates in many regions and led to higher costs for producing transgenic plants. Significant resources are allocated to strategies preventing transgene escape into the environment and addressing public safety concerns (Schutte et al., 2017). Thus, discovering naturally occurring glyphosate-tolerant mutations and introducing them into crop-specific EPSPS genes to develop glyphosate-tolerant crops is urgently needed to promote sustainable agriculture. At the same time, an extensive and thorough assessment of glyphosate’s effects on the environment and human health is required (Noack et al., 2024). Site-directed mutagenesis is a technique employed to enhance the aroA enzyme for the purpose of conferring resistance to glyphosate. However, it should be noted that the mutations introduced for glyphosate tolerance may result in a reduction or alteration of the binding affinity for S3P and PEP, as well as an impact on the enzyme’s catalytic efficiency, due to changes in its three-dimensional structure. It is therefore essential to explore naturally occurring mutations for glyphosate tolerance found in weed species and incorporate these into native aroA enzymes. This approach aims to achieve field-level glyphosate tolerance without compromising the EPSPS enzyme’s affinity for PEP or S3P.

The glyphosate resistance amino acid substitution mutation P106S was first identified in *E. indica (* Ng and Wickneswari, 2003). Additionally, several other P-106 substitution mutations to T, A, or L have been reported in glyphosate-resistant populations of *L. rigidum (* Zhou et al., 2006). These findings suggest that the high incidence of the P-106 substitution mutation to S, T, A, or L occurs in the EPSPS enzyme of bacteria and plants, indicating that P-106 might be a common mutation point for glyphosate resistance (Funke et al., 2009). The amino acid substitution T102I (corresponding to T173I in rice) alone provided strong glyphosate resistance; however, this mutation reduced the catalytic binding affinity for the PEP substrate (Funke et al., 2009). Another study reported that the T to I mutation (T97I) variant offers relatively less resistance to glyphosate, and the presence of the P101S mutation significantly reduces PEP affinity (Funke et al., 2009; Pollegioni et al., 2011). Notably, the combination of adjacent mutations at amino acid positions 106 and 102 in the active site of E. coli EPSPS (corresponding to T173I and P177S in rice) constitute the only combination of mutations that results in minor conformational changes, preventing glyphosate from binding to the EPSPS enzyme while maintaining intact PEP binding efficiency (Funke et al., 2009). Supporting this, the double substitution mutation (TIPS-NtEPSPS) provided a higher level (41.6 mM) of glyphosate tolerance compared to the single (P180S-NtEPSPS) substitution mutation (**Figure 4A**). Additionally, TIPS mutations in plants neutralized the negative effects of glyphosate.

To validate the function of a candidate gene, the use of transgenic platforms is the best approach. In the present study, the TIPS-NtEPSPS and P180S-NtEPSPS overexpressing tobacco lines under CaMV35S promoters were successfully generated. Among the constitutive promoters, the CaMV35S viral promoter has been among the most widely used promoters in basic research and in the development of transgenic plants due to the high levels of transgene expression in dicots. Attempts have been made to engineer rice plants for glyphosate resistance by overexpressing the aroA gene from different organisms, such as *M. domestica* (Tian et al., 2013)and *Janibacter* spp (Yi et al., 2016). The results from the present study showed that, compared with P180S-NtEPSPS-expressing tobacco lines, TIPS-NtEPSPS tobacco lines showed high degree of glyphosate tolerance (**Figures 3**, **4**). It has been reported that glyphosate application causes ROS accumulation (Ahsan et al., 2008), the development of glyphosate resistance is closely linked to the activation of the antioxidant system in plants (Maroli et al., 2015). Consistent with previous reports, the notable rise in the antioxidant enzyme activity of the transgenic lines in this experiment in response to glyphosate application may be the primary mechanism by which the resistance is developed (**Figures 4C**).

According to the previous study, EPSPS-gene overexpression is not involved in glyphosate resistance compared with mutant EPSPS-gene overexpression, such as *Tridax procumbens (* Li et al., 2018)and horseweed (Beres, 2019), we did not carry out the study of 35S promoter-driven EPSPS overexpression material in this experiment, however the increase in EPSPS copies may also contribute to tobacco’s herbicide resistance, hence it is necessary to carry out studies on the dose effect of the EPSPS gene in the future.

Crop rotation is a common planting practice in tobacco cultivation. However, the use of herbicides presents a considerable challenge in the context of rice-tobacco rotation. A crop-specific TIPS mutation in the class I aroA gene of rice and its overexpression have the potential to substantially improve the field-level glyphosate tolerance in rice. The plants demonstrated tolerance to up to 16.8 mM (Achary et al., 2020). Tobacco is highly sensitive to glyphosate, which causes severe injury to plants. Our results revealed that, compared to WT lines, TIPS-NtEPSPS lines exhibited a greater degree of tolerance to different doses of glyphosate, withstanding up to 10 μM in Petri dish conditions and 41.6 mM in pot conditions (**Figures 3A**, **4A**). The WT plants died 14 days after glyphosate treatment at a dose of 10 μM. This level of tolerance is superior to previous reports involving the transgenic overexpression of several class II aroA genes in tobacco (Yan et al., 2011).

The occurrence of multiple mutations in a single gene against a particular herbicide provides a significant advantage to plants in adapting to that herbicide, thereby representing a primary cause of the evolution of superweeds. In most cases, the evolution of multiple point mutations in a single allele occurs through recombination between natural plant populations that harbor single point mutations. This experiment provides a solid foundation for our gene editing in the future to produce non-transgenic herbicide-resistant tobacco materials using our developed efficient gene editing tools (Li et al., 2025).

The adoption of highly glyphosate-tolerant plants containing the TIPS mutation in the EPSPS gene in modern agriculture will be advantageous, as it will facilitate the application of high doses of glyphosate under field conditions for the control of all weed species. The application of higher doses of glyphosate will not only facilitate the effective control of weeds but will also prevent the evolution of weeds that have developed single glyphosate-tolerant point mutations, as these confer lower resistance to glyphosate.

## 4 Materials and methods

### 4.1 Experimental materials

#### 4.1.1 Plant materials and herbicides

Tobacco materials used for genetic transformation were *Nicotiana tabacum L.* cv. ‘Honghuadajinyuan’ (HD)from Institute of Tobacco Research, Chinese Academy of Agricultural Sciences (CAAS). The herbicide formulation used was aqueous 41% glyphosate isopropyl amine salt (Monsanto).

#### 4.1.2 Vectors and strains

PBI121 and pCAMBIA1305 were kept by Institute of Tobacco Research, Chinese Academy of Agricultural Sciences (CAAS), and the strains of Agrobacterium tumefaciens EHA105 and Escherichia coli DH5α, which were products of Dalian Baosheng Biological Company.

#### 4.1.3 Gene source

According to the cds sequence of EPSPS gene (protein ID: NP_001312842) of tobacco (*Nicotiana tabacum L.*) in NCBI database, the primers EPSPS-cds For and EPSPS-cds Rev (**Supplementary Table S2**) were designed to amplify the NtEPSPS gene, and the NtEPSPS gene was amplified by pcr primer design (EPSPS -T/I-P/S mutant and EPSPS-P180S mutant) to introduce single base mutations to obtain P180S-NtEPSPS and TIPS-NtEPSPS.

### 4.2 Experimental methods

#### 4.2.1 Molecular docking of NtEPSPS protein

Online comparison analysis was performed using NCBI Blast to select homologous protein sequences with high similarity, and the phylogenetic tree was constructed using MAGA 11.0 software; multiple sequence comparison was performed using DNAMAN. The molecular formula of glyphosate was obtained by searching PubChem database (https://pubchem.ncbi.nlm.nih.gov/). The small molecules were hydrogenated and charged in Auto Dock and predicted by AlphaFold (https://colab.research.google.com/github/sokrypton/ColabFold/blob/main/AlphaFold2.ipynb) protein tertiary structures of NtEPSPS, P180S-NtEPSPS and TIPS-NtEPSPS downloaded the pdb format files, deleted the excess water molecules in PyMOL, and opened the three proteins and glyphosate in Auto Dock via ZBH - Centre for Bioinformatics online platform (Zentrum für Bioinformatic: Universität Hamburg - Proteins Plus Server) to determine the coordinates of the binding site of the protein receptor, generate the docking box, and perform docking. Amino acids of protein and small molecule interaction sites were visualized.

#### 4.2.2 Plant expression vector construction

The genes was separately cloned into restriction enzymes BamH I and Sac I site of the pBI121 vector using Clon Express II One Step Cloning Kit to obtain the recombinant vectors pBI121 (P180S-NtEPSPS) and pBI121 (TIPS-NtEPSPS). The plant expression vectors pBI121 (P180S-NtEPSPS) and pBI121 (TIPS-NtEPSPS) were transferred to Agrobacterium tumefaciens EHA105 using the CaCl2 method, and the transformed Agrobacterium strains were stored at -80°C for later use. The pCAMBIA1305 was constructed in the same way as described above. The digestion sites were Xba I and Nco I.

#### 4.2.3 Agrobacterium-mediated genetic transformation and PCR assay of transformed plants

Tobacco (*Nicotiana tabacum L.* cv. ‘Honghuadajinyuan’) leaves in good condition were used for *Agrobacterium*-mediated genetic transformation (Lim et al., 2022) 10 μM glyphosate was used as a screening agent during the screening of resistant healing tissues. A small amount of genomic DNA was extracted from the transformed plants by the modified CTAB method (Carey et al., 2023). The extracted DNA was used as a template for PCR reaction to detect the target genes P180S-NtEPSPS and TIPS-NtEPSPS in the transformed plants, and the primers used for the detection of the target genes are shown in **Supplementary Table S2**. The PCR products were detected by 1.2% agarose gel electrophoresis.

#### 4.2.4 Transient expression in *Nicotiana benthamiana* and confocal microscopy

Transformed Agrobacterium tumefaciens strain EHA105 was grown overnight in liquid LB medium containing 25 μg/mL rifampin and 50 μg/mL kanamycin. Cells were washed and resuspended in infiltration solution (10 mM MgCl2,10mM MES, 100 μM acetosyringone), and diluted to an OD600 of 0.6-0.8, before mixing the transformed cells in the combinations to be tested. N. benthamiana leaves were infiltrated with the A. tumefaciens mix using a needleless syringe. Fluorescence was monitored approximately 48 h after infiltration.

A Leica SP5 confocal laser-scanning microscope with a 20 × 0.7 numerical aperture water-immersion objective was used to examine the lower epidermis of the infiltrated tobacco leaves. The complemented GFP fluorescence was excited at 488 nm and emission was detected at 515–540 nm. The gain was fixed in all samples to ensure that the emission intensity was comparable.

#### 4.2.5 Subcellular localization

the *NtEPSPS* open reading frames were inserted into pCAMBIA1305 to obtain the NtEPSPS-GFP constructs. The fusion constructs were introduced into *A. tumefaciens* strain EHA105 cells for the transformation of tobacco leaves via infiltration. After 72 hours incubation, the leaves were examined for GFP signals using the FluoView FV1000 confocal microscope (Olympus, Japan). The leaves co-transformed with the pCAMBIA1305 empty vector served as the negative control.

#### 4.2.6 RNA extraction and real time PCR of transgenic lines

Total RNA was isolated from 100 mg frozen tissue using reagent kit (QIAGEN, Germany). After determining the RNA quantity and purity using the Nanodrop ND-2000 spectrophotometer (Nanodrop Technologies, Wilmington, DE, USA), 5 mg RNA (50-μl reaction volume) were extracted. And AMV reverse transcriptase (Takara Biotechnology, Japan) were used for the first-strand cDNA synthesis (reverse transcription), which was completed at 42°C (1.5 μm), which was completed at 42°C for 1 h.

Total RNA was reverse transcribed using the Revert Aid™ First-Strand cDNA Synthesis Kit (MBI Fermentas, Canada). The synthesized cDNA along with TB Green™ Premix Ex Taq™ II (Tli RNaseH Plus; Takara, Japan) and candidate gene-specific primers (**Supplementary Table S2**), which were designed using Primer3Plus (http://primer3plus.com/cgi-bin/dev/primer3plus.cgi), were used for the qRT-PCR analysis performed using the 7500 fast Real-Time PCR System (Applied Biosystems, Foster City, CA, USA). Three fully independent biological replicates were prepared for the qRT-PCR runs conducted in triplicate, calculated using the 2^-ΔΔCt^ method (Damgaard and Treebak, 2022).

#### 4.2.7 Medium test of T1 generation transgenic lines

The sterilized seeds of T1 generation strains were divided into two parts, one part was inoculated into MS medium containing 0 mg/L glyphosate, and the other part was inoculated into medium containing 10 μM glyphosate, and incubated at 28°C, 16 h light/8 h dark for 14 d. Root lengths and the leaf areas of the first and second leaf positions of each lines were analyzed using ImageJ (Rueden et al., 2017).

#### 4.2.8 T1 generation transgenic strains in pot experiment

The agronomic traits of the candidate transgenic lines and HD(*Nicotiana tabacum L.* cv. ‘Honghuadajinyuan’)were further examined. The test materials were simultaneously sown and transplanted into 30 cm pots, with 60 plants transplanted in each treatment and the same water and fertilizer management. The T1 generation candidate transgenic lines were treated with two different doses of glyphosate in the field to detect glyphosate resistance. The two glyphosate doses tested were 0 and 41.6 mM, and each treatment was repeated three times. The glyphosate treatment process was as follows: 45 days after sowing, the first glyphosate treatment was applied to the test materials at the seedling stage; after 15 days of treatment, 20 tobacco plants were randomly selected from each treatment and transplanted into pots; 14 days after transplanting, the second glyphosate treatment was applied. The test materials were examined for aboveground fresh weight, underground dry weight, and antioxidant enzyme activities (SOD, POD, and APX) (Helander et al., 2012; Bhagat et al., 2023).

#### 4.2.9 Determination of glyphosate residue in transgenic lines

The T1 generation of the overexpressed material was planted in the field, with three replicates of 10 plants each, and treated with 41.6 mM glyphosate twice at the seedling and transplanting stages. Fifteen days after spraying at transplanting stage, whole plant tissues from five plants of each line were taken, and the plant surface was rinsed clean, stored on dry ice, and sent to Merieux Anchor Strength Commodity Inspection Ltd (Qingdao, China). Glyphosate residues were detected by the method SS/CHN/SOP/4086-01, which is an in-house method optimized according to the method SN/T 1923-2007.

#### 4.2.10 Assay method of superoxide dismutase activity

SOD activity was determined spectrophotometrically based on the inhibition of nitro blue tetrazolium (NBT) reduction at 450 nm. Leaf tissue (0.1 g) was homogenized in 1 mL of extraction buffer (50 mM phosphate buffer, pH 7.8) on ice, followed by centrifugation at 8000 × g for 10 minutes at 4°C. The resulting supernatant was retained for immediate analysis.

The reaction mixture consisted of 50 mM phosphate buffer (pH 7.8), 0.1 mM EDTA, 13 mM methionine, 75 µM NBT, and 2 µM riboflavin. The reaction was carried out at 37°C for 30 minutes, and absorbance at 450 nm was measured using a spectrophotometer preheated for 30 minutes. Percent inhibition of NBT reduction was calculated using the following formula:


$$\begin{array}{c} \text{Percent inhibition}\\ =\\ \left(\Delta \text{A\_blank}-\Delta \text{A\_determination}\right)/\Delta \text{A\_blank}\times 100\% \end{array}$$

SOD activity (U/mg protein) was calculated using the formula:


$$\begin{array}{c} \text{SOD activity}=11.11\times \text{Percent inhibition}/\left(1-\text{Percent inhibition}\right)\\ ÷\text{C\_pr}\times \text{F} \end{array}$$

where C_pr is the protein concentration (mg/mL), and F is the dilution factor.

#### 4.2.11 Assay method of peroxidase activity

POD activity was measured by monitoring the increase in absorbance at 470 nm due to the oxidation of guaiacol. Leaf tissue (0.1 g) was homogenized in 1 mL of extraction buffer (50 mM phosphate buffer, pH 6.0) on ice, followed by centrifugation at 8000 × g for 10 minutes at 4°C. The supernatant was used immediately for analysis.

The reaction mixture consisted of 50 mM phosphate buffer (pH 6.0), 10 mM guaiacol, and 20 mM H2O2. The reaction was initiated by adding the enzyme extract, and absorbance was recorded at 470 nm at 30 seconds (A1) and 90 seconds (A2). The change in absorbance (ΔA) was calculated as A2 - A1.

POD activity (U/mg protein) was calculated using the formula:


$$\begin{array}{c} \text{POD activity}=\left(\Delta \text{A}\times \text{V}\_\text{total}\right)/\left(\text{V}\_\text{sample}\times \text{C}\_\text{pr}\times 0.01\times \text{T}\right)\\ =7133\times \Delta \text{A}/\text{C}\_\text{pr} \end{array}$$

where V_total is the total reaction volume, V_sample is the sample volume, C_pr is the protein concentration (mg/mL), and T is the time interval.

#### 4.2.12 Assay method of ascorbate peroxidase activity

APX activity was determined by measuring the decrease in absorbance at 290 nm due to the oxidation of ascorbic acid (AsA). Leaf tissue (0.1 g) was homogenized in 1 mL of extraction buffer (50 mM phosphate buffer, pH 7.0, containing 1 mM EDTA and 2 mM AsA) on ice. The homogenate was centrifuged at 13,000 × g for 10 minutes at 4°C, and the supernatant was collected for immediate analysis.

The reaction mixture contained 50 mM phosphate buffer (pH 7.0), 0.5 mM AsA, and 0.1 mM H2O2. Absorbance was recorded at 290 nm at 10 seconds (A1) and 2 minutes 10 seconds (A2), and the change in absorbance (ΔA) was calculated as A2 - A1.

APX activity (U/mg protein) was calculated using the following formula:


APX activity=[(ΔA_assay tube-ΔA_blank tube)/(ϵ×d)]×(V _total×10^6)/(C_pr×V _sample×T)                      =1.79×(ΔA_assay tube−ΔA_blank tube)/C_pr

where ϵ is the extinction coefficient of AsA (2.8 mM^-1^ cm^-1^), d is the path length of the cuvette (1 cm), C_pr is the protein concentration (mg/mL), and T is the reaction time.

## 5 Conclusion

Our research demonstrated the function of EPSPS with two different mutations (T1766I + P180S; TIPS -NtEPSPS and P180S; P180S-NtEPSPS) and the effects of such mutations on tobacco plant growth at the biochemical and physiological levels. Overexpression of TIPS-NtEPSPS conferred greater tolerance to glyphosate (up to four times the recommended dose) without compromising plant fitness in controlled environments. The present results are important for the design of resistance management practices that can minimize the evolution of glyphosate resistance in tobacco.

## Data Availability

The raw data supporting the conclusions of this article will be made available by the authors, without undue reservation.

## Supplementary material

Supplementary Table 1 List of primers used in the study.

Supplementary Table 2 Similarity percentage of EPSPS protein sequences among various EPSPS enzymes from different organisms. Sequence identity of class I EPSPS enzymes belonging to Rigid ryegrass (82.39%), Rice (82.86%), Rubber tree(84.74%), wheat(83.1%), cotton(87.04%), pepper(90.73%), maize(83.64%), butter weed(87.3%), Arabidopsis thaliana(83.84%), goosegrass(82.02%), soybean(85.32%) and, 12.33% identity with the class II enzymes Agrobacterium.
