# Correction: Exploring the potential role of EPSPS mutations for enhanced glyphosate resistance in *Nicotiana tabacum*

**DOI:** 10.3389/fpls.2025.1653403

**Published:** 2025-07-29

**Authors:** Bingjie Li, Chen Chen, Mengmeng Cui, Yuhe Sun, Jing Lv, Changbo Dai

**Affiliations:** ^1^ Tobacco Research Institute, Chinese Academy of Agricultural Sciences, Qingdao, China; ^2^ Graduate School of Chinese Academy of Agricultural Sciences, Beijing, China

**Keywords:** glyphosate, herbicide resistance, EPSP synthase, tobacco, gene editing

In the published article, there was an error in the **Funding** statement. The funding number “110202101036 (JY-06)” was incorrect and is corrected to “110202201006(JY-06)”. The correct **Funding** statement appears below.

“The author(s) declare financial support was received for the research, authorship, and/or publication of this article. The Agricultural Science and Technology Innovation Program (ASTIP-TRIC02, ASTIP-TRIC-ZD03, ASTIP-TRIC-ZD05). China Tobacco Genome Project (110202101036 (JY-13), 110202201006(JY-06)). Natural Science Foundation of Shandong Province (ZR2023QC159). Key Laboratory of Tobacco Pest Monitoring and Integrated Management (KLTPMIMT2022-03).”

The original article has been updated.

