# Supplementary material for: Exploring the potential role of EPSPS mutations for enhanced glyphosate resistance in Nicotiana tabacum
Source: Front Plant Sci. 2025 Feb 10;16:1516963. doi: 10.3389/fpls.2025.1516963 (PMC11847837; doi:10.3389/fpls.2025.1516963)
Supplement: Supplementary Table 1 — List of primers used in the study. [file Table1.docx]

Tables S1 Similarity percentage of EPSPS protein sequences among various EPSPS enzymes from different organisms. Sequence identity of class I EPSPS enzymes belonging to Rigid ryegrass （82.39%），Rice （82.86%），Rubber tree（84.74%） ，wheat（83.1%） ，cotton（87.04%），pepper（90.73%），maize（83.64%），butter weed（87.3%），Arabidopsis thaliana（83.84%），goosegrass（82.02%）， soybean（85.32%） and, 12.33% identity with the class II enzymes Agrobacterium.

| **Species** | **Accession no** | **Protein length** | **protein percentage identities %** |
| --- | --- | --- | --- |
| Tobacco | NP_001312842 | 518 | 100 |
| Tobacco | XP_009802118 | 518 | 99.61 |
| Rigid ryegrass | XP_047075216 | 510 | 82.39 |
| Rice | XP_015643046 | 515 | 82.86 |
| Rubber tree | XP_057998781 | 522 | 84.74 |
| wheat | XP_044425014 | 510 | 83.1 |
| Cotton | XP_016735170 | 521 | 87.04 |
| Pepper | XP_016575220 | 521 | 90.73 |
| Maize | XP_008659331 | 506 | 83.64 |
| Butter weed | XP_043620984. | 519 | 87.3 |
| Arabidopsis | NP_182055 | 520 | 83.84 |
| Goosegrass | AAN63156 | 445 | 82.02 |
| AgrobactriumCP4 | Q9R4E4 | 455 | 12.33 |
| Soybean | XP_003521857 | 526 | 85.32 |
