# Supplementary material for: Exploring the potential role of EPSPS mutations for enhanced glyphosate resistance in Nicotiana tabacum
Source: Front Plant Sci. 2025 Feb 10;16:1516963. doi: 10.3389/fpls.2025.1516963 (PMC11847837; doi:10.3389/fpls.2025.1516963)
Supplement: Supplementary Table 2 — Similarity percentage of EPSPS protein sequences among various EPSPS enzymes from different organisms. Sequence identity of class I EPSPS enzymes belonging to Rigid ryegrass (82.39%), Rice (82.86%), Rubber tree(84.74%), wheat(83.1%), cotton(87.04%), pepper(90.73%), maize(83.64%), butter weed(87.3%), Arabidopsis thaliana(83.84%), goosegrass(82.02%), soybean(85.32%) and, 12.33% identity with the class II enzymes Agrobacterium. [file Table2.docx]

Table S2 List of primers used in the study.

| **Primer name** | **Primer Sequence 5‘---------3’** |
| --- | --- |
| EPSPS-cds For | ATGGCACAGATTAGCAGCATGG |
| EPSPS-T/I-P/S mutant For | GGAATAGCAATGCGGTCATTGACGGCAGCAGTTAC |
| EPSPS-T/I-P/S mutant Rev | TGACCGCATTGCTATTCCTGCATTTCCAAGGAATA |
| EPSPS-P180S mutant For | GGAACAGCAATGCGGTCATTGACGGCAGCAGTTAC |
| EPSPS-P180S mutant Rev | TGACCGCATTGCTGTTCCTGCATTTCCAAGGAATA |
| EPSPS-cds Rev | TTAATGCTTGGAGTACTGCTGGAGAA |
| 1305-EPSPS-GFP(XbaI)For | AAGTCCGGAGCTAGCTCTAGAATGGCACAGATTAGCAGCATGG |
| 1305-EPSPS-GFP(NcoI) Rev | CCTCGCCCTTGCTCACCATGGATGCTTGGAGTACTGCTGGAGAA |
| EPSPS-T/I-P/S mutant -qrt For | ATGCAGGAATAGCAATGCGGTCATT |
| EPSPS-T/I-P/S mutant -qrt Rev | GGAAGACCTCCCTTGCTGACTATCC |
| EPSPS-P180S mutant -qrt For | ATGCGGTCATTGACGGCAGCAGTTA |
| EPSPS-P180S mutant -qrt Rev | ACAATTTGTACCAAGGAAACAATCA |
| Actin-F | CTGACATCTCCCGCACTCTTA |
| Actin-R | CATAGTCCATTCGTAGTTGAGCA |
